# Prospective systematic review registration: perspective from the Guidelines International Network (G-I-N)

**DOI:** 10.1186/2046-4053-1-3

**Published:** 2012-02-09

**Authors:** Philip Van der Wees, Amir Qaseem, Minna Kaila, Guenter Ollenschlaeger, Richard Rosenfeld

**Affiliations:** 1 Scientific Institute for Quality of Healthcare, Radboud University Nijmegen Medical Center, PO Box 9101, 6500 HB Nijmegen, the Netherlands; 2 School CAPHRI, Maastricht University, PO BOX 616, 6200 MD Maastricht, the Netherlands; 3 Royal Dutch Society for Physical Therapy, PO BOX 248, 3800 AE Amersfoort, the Netherlands; 4 Department of Health Care Policy, Harvard Medical School, 180 Longwood Avenue, Boston, MA 02115, USA; 5 Department of Clinical Policy, American College of Physicians, 190 North Independence Mall West, Philadelphia, PA 19106, USA; 6 Hjelt Institute, University of Helsinki, PO Box 40, 00014 Helsinki, Finland; 7 German Agency for Quality in Medicine AEZQ, Strasse des 17. Juni 106-108, 10623 Berlin, Germany; 8 Department of Otolaryngology, State University of New York Downstate Medical Center and Long Island College Hospital, 134 Atlantic Avenue, Brooklyn, NY 11201, USA

**Keywords:** guidelines, reviews, systematic, methods

## Abstract

Clinical practice and public health guidelines are important tools for translating research findings into practice with the aim of assisting health practitioners as well as patients and consumers in health behavior and healthcare decision-making. Numerous programs for guideline development exist around the world, with growing international collaboration to improve their quality. One of the key features in developing trustworthy guidelines is that recommendations should be based on high-quality systematic reviews of the best available evidence. The review process used by guideline developers to identify and grade relevant evidence for developing recommendations should be systematic, transparent and unbiased. In this paper, we provide an overview of current international developments in the field of practice guidelines and methods to develop guidelines, with a specific focus on the role of systematic reviews. The Guidelines International Network (G-I-N) aims to stimulate collaboration between guideline developers and systematic reviewers to optimize the use of available evidence in guideline development and to increase efficiency in the guideline development process. Considering the significant benefit of systematic reviews for the guideline community, the G-I-N Board of Trustees supports the international prospective register of systematic reviews (PROSPERO) initiative. G-I-N also recently launched a Data Extraction Resource (GINDER) to present and share data extracted from individual studies in a standardized template. PROSPERO and GINDER are complementary tools to enhance collaboration between guideline developers and systematic reviewers to allow for alignment of activities and a reduction in duplication of effort.

## Background

Clinical practice and public health guidelines are "systematically developed statements designed to help practitioners and patients to make decisions about appropriate healthcare for specific circumstances" [1] (p. 38). The Institute of Medicine recently updated this early definition to underscore the importance of systematic review of the evidence and both benefits and harms assessment as essential characteristics of practice guidelines: "Clinical practice guidelines are statements that include recommendations intended to optimize patient care, that are informed by a systematic review of evidence and an assessment of the benefits and harms of alternative care options" [2] (p. 4). In healthcare services, clinical guidelines are considered important instruments with which to improve and manage the care process [3-6]. Important goals in developing and implementing guidelines are higher quality and improved cost-effectiveness of interventions, ideally resulting in improved health outcomes [7,8].

The growing body of knowledge in the field of clinical guidelines has provided opportunities for international collaboration. In 2002, the Guidelines International Network (G-I-N) was founded to provide a network and partnerships for guideline organizations, implementers, researchers and other stakeholders in healthcare [9]. G-I-N seeks to improve the quality of health care by promoting the systematic development of guidelines and their application to practice.

One of the key features in developing guidelines is to ensure that guideline recommendations are based, as much as possible, on high-quality systematic reviews of the best available evidence. Similarly, the review process used by guideline developers to identify and grade relevant evidence for developing recommendations should be systematic, transparent and unbiased. The medical literature is expanding quickly [10], and ongoing collaboration between guideline developers and systematic reviewers is needed to maximize the use of the best available evidence to support guideline recommendations. The international prospective register of systematic reviews (PROSPERO) is a promising new initiative whose purpose is to stimulate collaboration, reduce duplication of research efforts and improve quality [11].

In this paper, we introduce the international guideline community and describe current methods for developing guidelines with a specific focus on the role of systematic reviews in guideline development. The aim of this paper is to create common ground for guideline developers and systematic reviewers on which to base their collaboration.

## The Guidelines International Network (G-I-N)

G-I-N is a global network which comprises 93 organizations and 89 individuals as members representing 46 countries from all continents (as of January 2012). The network supports evidence-based healthcare and improvement of health outcomes throughout the world. The specific aims of G-I-N are to promote best practices and reduce duplication of research efforts by improving the efficiency and effectiveness of evidence-based guideline development, adaptation, dissemination and implementation. One of the main assets of G-I-N is its International Guideline Library. The library contains more than 7,400 documents (as of January 2012) and includes guidelines (*N *= 3,636), evidence reports, methodologies and other related documents developed or endorsed by G-I-N member organizations [12].

A recent, important new product of G-I-N in relation to systematic reviews has been developed by G-I-N's Evidence Tables Working Group. This group has developed a minimum data set that can be used to summarize published studies. A template for intervention studies has been developed [13] in addition to a template for diagnostic studies. Templates for healthcare economic and prognostic studies are in preparation. The templates allow for consistent comparison across studies and to inform a group process in synthesizing evidence. On this basis, a resource has been developed to allow reviewers to present data extracted from individual studies using a standardized template (called a "summary" for short). This resource forms the foundation for development of evidence tables which summarize data based on a defined question. The summaries can then be used by G-I-N members in their guideline development process to populate their evidence tables using the data directly as presented in the original research articles or modified according to their specific needs. The registry was launched in August 2011 at the Eighth G-I-N Conference in Seoul under the acronym of GINDER: G-I-N Data Extraction Resource [12].

## Methodology for guideline development

Methods for guideline development have been harmonized to a certain degree [14,15]. Core elements of the process are formulating the relevant healthcare clinical questions, identifying relevant outcomes, systematically identifying and summarizing the relevant evidence, synthesizing the evidence by evaluation of its quality and formulating recommendations for daily practice or healthcare policy. The steps in guideline development are given in Table 1.

**Table 1:** Elements in guideline development^a^

Elements	Specification
1. Organization and structure	National or professional coordinated program. Development by (multidisciplinary) working groups, including healthcare practitioners, systematic reviewers and/or methodologists and patients.
2. Preparation	Definition of scope and objectives. Formulation of health and clinical questions and patient-important outcomes. Specification of targeted patient or population groups and intended users.
3. Development process	Identification of evidence via systematic literature search. Assessment and synthesis of evidence. Translation of evidence into recommendations for daily practice and health policy.
4. Validation	External review of draft guidelines by peers and stakeholders, as well as, if feasible, by field-testing.
5. Dissemination and implementation	Publication on paper and/or electronically via website or journal. Further implementation via tailored strategies to promote the actual use of the guideline.
6. Evaluation and revision	Regular update based on scheduled review (every 3 to 5 years) and updating procedure.

^a^Source: Van der Wees *et al. *[15].

A standard guideline development process is essential to ensuring that developers publish valid, reliable guidelines that can be used in their research. The Appraisal of Guidelines, Research and Evaluation (AGREE II) instrument is a valuable tool with which to analyze the rigor of development of a good guideline [16], and standards for guideline development have been developed at the national level, such as the recently published standards for trustworthy guidelines issued by the Institute of Medicine [2]. Thus far, however, the guideline community has not established a common set of internationally recognized standards to help improve the development of high quality guidelines. International standards will facilitate information sharing and adaptation to reduce duplication of efforts and support initiatives for the development of high-quality national or local guidelines. The G-I-N Board of Trustees recently prepared a position paper with key components describing how to develop and evaluate quality clinical practice guidelines (Qaseem A. et al, Guidelines International Network: Towards International Standards for Clinical Practice Guidelines, Annals of Internal Medicine 2012;156 (forthcoming)). This position paper should contribute to further harmonization of the guideline development process and stimulate the development of international guideline standards.

## The role of systematic reviews in guideline development

Recommendations in guidelines are typically derived from a set of key questions for prevention, diagnosis and/or treatment for the target group of patients. Key questions should include elements that will guide the literature review process: the familiar patient problem or population, intervention, comparison and outcomes (PICO) format [17]. Other issues to be considered are the importance of the effectiveness of interventions by estimating the effect sizes for the relevant outcomes and whether the desirable effects (benefits) outweigh the undesirable effects (harms) [18]. Weighing benefits and harms also requires the collection of empirical data or qualitative information about the values and preferences of patients.

The literature review to identify and summarize the evidence must be carried out in such a way that the potential for any bias is minimized. Some manuals of national guideline programs include the production of full systematic reviews, whereas others primarily identify existing systematic reviews and conduct additional reviews if necessary and feasible [19-23]. Depending on the number of questions, multiple systematic reviews may be required to inform a guideline. A recent editorial published in the *Cochrane Database of Systematic Reviews *shows that the National Institute of Health and Clinical Excellence frequently uses the Cochrane reviews in issuing guidelines, although the authors of the editorial advocate better use of Cochrane reviews, as well as more collaboration between groups, in developing guidelines [24]. Another option used by guideline developers is to identify evidence in existing guidelines, because guideline databases are growing [12,25].

Systematic reviews intended to inform the development of clinical guidelines should follow principles outlined in published checklists such as the PRISMA statement for transparent reporting of systematic reviews and meta-analyses [26]. The Institute of Medicine recently published standards for systematic reviews, which include a standard for interaction between guideline developers and systematic reviewers [27]. Use of an explicit and transparent strategy to evaluate evidence helps users to interpret the relative strength of the evidence [28,29]. Identified studies are systematically appraised by assessing the methodological quality and relevance of the clinical context in the study. On the basis of the quality of individual studies, an overall synthesis of the evidence will result in evidence statements, usually expressed as levels of evidence. The Grading of Recommendations Assessment, Development and Evaluation (GRADE) approach is increasingly being adopted by guideline development organizations worldwide [18]. The use of observational data has been advocated to create evidence for everyday practice needs, and adaptive approaches have been proposed to synthesize evidence using outcomes of both randomized trials and observational research [30,31]. International collaboration to address these issues is important for building a body of knowledge and standards in guideline development.

## Translating evidence into recommendations for clinical practice

Once the evidence is identified and appraised, the guideline development group must consider its relevance and applicability to practice, including anticipated benefits and harms, to formulate recommendations that can be put into action [32]. Guideline developers must make a considered judgment about the generalizability, applicability, consistency and clinical impact of the evidence to create a clear link between recommendations and the underlying evidence. This is a crucial part of the guideline development process in which local circumstances need to be taken into account. The strength of recommendations included in guidelines is usually subject to a grading system, such as the GRADE approach, that takes into account the quality of the evidence and the considered judgment of the guideline developers [18]. Transparency of the considered judgment of guideline development groups is important to understanding the arguments for specific recommendations. Guideline recommendations should be expressed in clear, unambiguous language to facilitate implementation [33].

## Perspective for collaboration

Because developing new systematic reviews and using existing ones are essential components of the guideline development process, there is clearly potential for better collaboration between guideline developers and systematic reviewers. Interaction between guideline developers and systematic reviewers is important for formulating PICO questions, conducting the review of the evidence, compiling the evidence tables and summarizing and presenting the evidence. Resource constraints on guideline development may limit the number of new systematic reviews that can actually be carried out in a guideline project. In addition, many systematic reviews do not meet the standards that are essential for use in developing a good guideline [2,27]. Collaborative approaches to further improvement of the quality of systematic reviews and the use of high-quality reviews in translating evidence into guideline recommendations are therefore essential.

Alderson and Tan [24] made a plea for better use of Cochrane reviews in guideline development, and PROSPERO may help to achieve that objective. A crucial factor in fostering better collaboration is alignment of activities at the very start of the process. Guideline developers should be aware of existing or expected systematic reviews to support their project, and systematic reviewers should be aware of the relevant healthcare or clinical questions in focusing their reviews. The prospective registration of systematic review protocols provides an excellent basis from which to seek alignment and create opportunities for including systematic reviewers as members of the guideline development group. Identification of upcoming or existing systematic reviews can reduce the workload and resources needed for guideline development. The participation of systematic reviewers in the guideline development process enhances the application of their research findings to practice and help bridge the gap between research and practice. The GINDER registry of evidence summaries can further enhance collaboration and reduce duplication of efforts.

G-I-N and PROSPERO have recognized the possibility of closer collaboration and have started to encourage their members and affiliates to work together. G-I-N stimulates its members to use PROSPERO in searching for published systematic reviews and systematic review protocols, and G-I-N promotes the registration of systematic reviews within guideline development projects. PROSPERO has created a link to G-I-N's website to raise awareness among systematic reviewers of the potential impact of their work on the development of guideline recommendations and to facilitate the use of GINDER.

## Conclusion

A registry of high-quality systematic reviews based on rigorous standards is a topic of international interest. We think that the international prospective register of systematic reviews is of significant value for the international guideline community. This paper summarizes the view of G-I-N's Board of Trustees that high-quality systematic reviews are essential for developing good clinical practice and public health guidelines and that G-I-N supports the PROSPERO initiative. PROSPERO and GINDER are important complementary tools with which to enhance collaboration between guideline developers and systematic reviewers and allow for alignment of activities and reduced duplication of efforts.

## Competing interests

The authors wrote this paper from the perspective as trustees of the G-I-N Board. The interest of G-I-N is to promote the importance of practice guidelines and to improve the efficiency and effectiveness of evidence-based guideline development, adaptation, dissemination and implementation. The authors had no financial competing interests in the writing of this paper.

## Authors' contributions

PvdW wrote the main body of the paper with the participation of AQ, MK, GO and RR. Members of the G-I-N 2011/12 Board of Trustees (DD, FF, FM, SP, DS, RS and JS) provided feedback on the draft paper and approved the final manuscript.

## References

[B1] Field MJ Lohr K Guidelines for Clinical Practice, from Development to Use 1992 Washington, DC: National Academies Press 25121254

[B2] Institute of Medicine Clinical Practice Guidelines We Can Trust 2011 Washington, DC: National Academies Press

[B3] Grimshaw J Freemantle N Wallace S Russell I Hurwitz B Watt I Long A Sheldon T Developing and implementing clinical practice guidelines Qual Health Care 1995 4 55 64 10142039 10.1136/qshc.4.1.55 PMC1055269

[B4] Grimshaw JM Russell IT Achieving health gain through clinical guidelines II: Ensuring guidelines change medical practice Qual Health Care 1994 3 45 52 10136260 10.1136/qshc.3.1.45 PMC1055182

[B5] Grol R Grimshaw J From best evidence to best practice: effective implementation of change in patients' care Lancet 2003 362 1225 1230 14568747 10.1016/S0140-6736(03)14546-1

[B6] Grol R Wensing M Eccles M Improving Patient Care: The Implementation of Change in Clinical Practice 2005 London: Elsevier

[B7] Grimshaw JM Thomas RE MacLennan G Fraser C Ramsay CR Vale L Whitty P Eccles MP Matowe L Shirran L Wensing M Dijkstra R Donaldson C Effectiveness and efficiency of guideline dissemination and implementation strategies Health Technol Assess 2004 8 iii-iv 1 72 10.3310/hta8060 14960256

[B8] Woolf SH Grol R Hutchinson A Eccles M Grimshaw J Clinical guidelines: potential benefits, limitations, and harms of clinical guidelines BMJ 1999 318 527 530 10024268 10.1136/bmj.318.7182.527 PMC1114973

[B9] Ollenschläger G Marshall C Qureshi S Rosenbrand K Burgers J Mäkelä M Slutsky J Board of Trustees 2002, Guidelines International Network (G-I-N) Improving the quality of health care: using international collaboration to inform guideline programmes by founding the Guidelines International Network (G-I-N) Qual Saf Health Care 2004 13 455 460 15576708 10.1136/qshc.2003.009761 PMC1743909

[B10] Druss BG Marcus SC Growth and decentralization of the medical literature: implications for evidence-based medicine J Med Libr Assoc 2005 93 499 501 16239948 PMC1250328

[B11] Booth A Clarke M Ghersi D Moher D Petticrew M Stewart L An international registry of systematic-review protocols Lancet 2011 377 108 109 20630580 10.1016/S0140-6736(10)60903-8

[B12] Guidelines International Network (G-I-N) International Guideline Library http://www.g-i-n.net/library/international-guidelines-library/

[B13] Mlika-Cabanne N Harbour R de Beer H Laurence M Cook R Twaddle S Guidelines International (GIN) Working Group on Evidence Tables Sharing hard labour: developing a standard template for data summaries in guideline development BMJ Qual Saf 2011 20 141 145 10.1136/bmjqs.2010.040923 21209129

[B14] Burgers JS Grol R Klazinga NS Mäkelä M Zaat J AGREE Collaboration: Towards evidence-based clinical practice: an international survey of 18 clinical guideline programs Int J Qual Health Care 2003 15 31 45 12630799 10.1093/intqhc/15.1.31

[B15] Van der Wees PJ Hendriks EJ Custers JW Burgers JS Dekker J de Bie RA Comparison of international guideline programs to evaluate and update the Dutch program for clinical guideline development in physical therapy BMC Health Serv Res 2007 7 191 18036215 10.1186/1472-6963-7-191 PMC2228296

[B16] Brouwers MC Kho ME Browman GP Burgers JS Cluzeau F Feder G Fervers B Graham ID Hanna SE Makarski J AGREE Next Steps Consortium Development of the AGREE II, part 1: performance, usefulness and areas for improvement CMAJ 2010 182 1045 1052 20513780 10.1503/cmaj.091714 PMC2900328

[B17] Sackett DL Straus SE Richardson WS Rosenberg W Haynes RB Evidence-Based Medicine: How to Practice and Teach EBM 2000 Edinburgh: Churchill Livingston

[B18] Guyatt GH Oxman AD Vist GE Kunz R Falck-Ytter Y Alonso-Coello P Schünemann HJ GRADE Working Group GRADE: an emerging consensus on rating quality of evidence and strength of recommendations BMJ 2008 336 924 926 18436948 10.1136/bmj.39489.470347.AD PMC2335261

[B19] German Medical Association, National Association of Statutory Health Insurance Physicians, Association of the Scientific Medical Societies National Program for Disease Management Guidelines: Methods Manual 2010 Berlin/Duesseldorf: Ärtzliches Zentrum für Qualität in der Medicine (ÄZQ)

[B20] Agency for Healthcare Research and Quality (AHRQ) U.S. Preventive Taskforce Procedure Manual 2008 Rockville, MD: AHRQ

[B21] CBO Evidence-Based richtlijnontwikkeling: Handleiding voor werkgroepleden 2007 Utrecht: Kwaliteitsinstituut voor de gezondheidszorg CBO

[B22] National Health and Medical Research Council (NHMRC) Procedures and Requirements for Meeting the 2011 NHMRC Standard for Clinical Practice Guidelines 2011 Canberra: NHMRC

[B23] National Institute for Health and Clinical Excellence (NICE) The Guidelines Manual 2009 London: NICE

[B24] Alderson P Tan T The use of Cochrane Reviews in NICE clinical guidelines Cochrane Database Syst Rev 2011 9 ED000032 21901736 10.1002/1312103.ED000032

[B25] Agency For Healthcare Research and Quality (AHRQ) National Guideline Clearinghouse http://www.guideline.gov/

[B26] Moher D Liberati A Tetzlaff J Altman DG PRISMA Group Preferred reporting items for systematic reviews and meta-analyses: the PRISMA statement Ann Intern Med 2009 151 264 269, W64 19622511 10.7326/0003-4819-151-4-200908180-00135

[B27] Institute of Medicine Finding What Works in Health Care: Standards for Systematic Reviews 2011 Washington, DC: Institute of Medicine 24983062

[B28] Ansari MT Tsertsvadze A Moher D Grading quality of evidence and strength of recommendations: a perspective PLoS Med 2009 6 e1000151 19753108 10.1371/journal.pmed.1000151 PMC2735783

[B29] Kavanagh BP The GRADE system for rating clinical guidelines PLoS Med 2009 6 e1000094 19753107 10.1371/journal.pmed.1000094 PMC2735782

[B30] Luce BR Kramer JM Goodman SN Connor JT Tunis S Whicher D Schwartz JS Rethinking randomized clinical trials for comparative effectiveness research: the need for transformational change Ann Intern Med 2009 151 206 209 19567619 10.7326/0003-4819-151-3-200908040-00126

[B31] Sox HC Greenfield S Comparative effectiveness research: a report from the Institute of Medicine Ann Intern Med 2009 151 203 205 19567618 10.7326/0003-4819-151-3-200908040-00125

[B32] Rosenfeld RM Shiffman RN Clinical practice guideline development manual: a quality-driven approach for translating evidence into action Otolaryngol Head Neck Surg 2009 140 6 Suppl 1 S1 S43 19464525 10.1016/j.otohns.2009.04.015 PMC2851142

[B33] Shiffman RN Dixon J Brandt C Essaihi A Hsiao A Michel G O'Connell R The GuideLine Implementability Appraisal (GLIA): development of an instrument to identify obstacles to guideline implementation BMC Med Inform Decis Mak 2005 5 23 16048653 10.1186/1472-6947-5-23 PMC1190181

